# Formation and Characterization of Stable TiO_2_/Cu_x_O-Based Solar Cells

**DOI:** 10.3390/ma16165683

**Published:** 2023-08-18

**Authors:** Grzegorz Wisz, Paulina Sawicka-Chudy, Maciej Sibiński, Rostyslav Yavorskyi, Mirosław Łabuz, Dariusz Płoch, Mariusz Bester

**Affiliations:** 1 Institute of Materials Engineering, College of Natural Sciences, University of Rzeszow, Pigonia 1, 35-310 Rzeszow, Poland; dploch@ur.edu.pl; 2 Department of Material and Environmental Technology, Tallinn University of Technology, Ehitajate tee 5, 19086 Tallinn, Estonia; maciej.sibinski@p.lodz.pl; 3 Department of Semiconductor and Optoelectronic Devices, Lodz University of Technology, Al. Politechniki 10, 93-590 Łódź, Poland; 4 Department of Physics and Chemistry of Solid State, Vasyl Stefanyk Precarpation National University, T. Shevchenko Str. 57, 76-018 Ivano-Frankivsk, Ukraine; r.yavorskyi@pnu.edu.ua; 5 Institute of Physics, College of Natural Sciences, University of Rzeszow, Pigonia 1, 35-310 Rzeszow, Poland; mlabuz@ur.edu.pl (M.Ł.); mbester@ur.edu.pl (M.B.)

**Keywords:** thin films, TiO_2_/Cu_x_O, solar cells, renewable energy, DC magnetron sputtering

## Abstract

According to increasing demand for energy, PV cells seem to be one of the best answers for human needs. Considering features such as availability, low production costs, high stability, etc., metal oxide semiconductors (MOS) are a focus of attention for many scientists. Amongst MOS, TiO_2_ and Cu_x_O seem to be promising materials for obtaining an effective photoconversion effect. In this paper, specific investigation, aimed at the manufacturing of the complete photovoltaic structure based on this concept is described in detail. A set of samples manufactured by DC magnetron sputtering, with various process parameters, is characterized by morphology comparison, layer structure and material composition investigation, and finally by the obtained photovoltaic parameters. Based on SEM studies, it was established that the films are deposited uniformly and complete their formation; without clearly defined faces, the conglomerates of the film grow individually. These are areas with a uniform structure and orientation of atoms. The sizes of conglomerates are in a normal direction range from 20 to 530 nm and increase with film thickness. The film thickness was in the range from 318 to 1654 nm, respectively. The I-V study confirms the photovoltaic behavior of thin film solar cells. The open-circuit voltage (V_oc_) and short-circuit current density (J_sc_) values of the photovoltaic devices ranged from 1.5 to 300 mV and from 0.45 to 7.26 µA/cm^3^, respectively, which corresponds to the maximum efficiency at the level of 0.01%. Specific analysis of the junction operation on the basis of characteristics flow, R_s_, and R_sh_ values is delivered.

## 1. Introduction

People always needed effective and easily accessible sources of energy to function. Over the centuries, and especially recently, such a demand evidently grew, and this trend will continue. Renewable sources, including solar energy converted into electricity using photovoltaic cells, have an increasing share in the energy market. Photovoltaic cells often have connotations with the silicon, while scientists are still looking for more cost-effective solutions, characterized by non-toxicity, stability, and availability.

One of the ways of development of photovoltaic cells concerns Cu_2_ZnSnS_4_ (CZTS) thin films [1,2,3,4,5,6,7]. CZTS has the band gap of ~1.50 eV, being a very close value to the best band gap required by a semiconductor solar cell (according to Shockley–Queisser theory) −1.4–1.45 eV [8,9]. Thanks to features such as the direct band gap and a high absorption coefficient, CZTS material can be applied as the absorption layer of thin film solar cells. Guo et al. [2] implemented Ti as an intermediate layer in the CZTS structure at the interface between Mo and Cu_2_ZnSnS_4_. The authors showed that such an implementation improved the Hall mobility, surface evenness, crystallinity, and absorptivity of CZTS, as well as inhibited the formation of voids at the interface between the Mo and Cu_2_ZnSnS_4_ layer. The authors showed also that the application of the Ti layer resulted in an increase of 32% of the open-circuit voltage of the CZTS as well as the increase of 57% in conversion efficiency. Lee et al. [4] investigated the FTO/TiO_2_/In_2_S_3_/Cu_2_ZnSnS_4_ cell with the spray deposition method and received the efficiency of 3.34%. The authors concluded, however, that the aqueous-based solution, which they used, could result in surface oxidation and limit grain growth, which leads to a low device performance. Satale et al. [5] considered a FTO/TiO_2_/CdS/CZTS/Au structure and showed that by optimizing the composition of Cu and Zn, it was possible to obtain the power conversion efficiency up to 1.04%. The authors made the CZTS film at the temp. 250 °C, without sulfurization, with CdS as an interface, and with TiO_2_ nanorod arrays processed hydrothermally. Khalil et al. [6] reported about CZTS fabricated on flexible Mo foil substrates with the electrodeposition–annealing method. The authors obtained 0.55% power conversion efficiency.

Promising materials for PV implementation are metal oxide (MO) semiconductors (MOS), which, thanks to features such as high sensitivity, low power consumption, and ease of synthesis, are widely investigated [10,11,12,13,14,15,16].

Very good representatives of MO are titanium dioxide (TiO_2_) and cupric oxide/cuprous oxide (CuO/Cu_2_O), as they are cheap materials, chemically neutral, with environmentally friendly resources and highly resistant to photocorrosion [17,18,19,20,21,22,23,24]. On the other hand, as the titanium dioxide has a bandgap of 2.96–3.2 eV [25,26,27] (depending on the TiO_2_ form), it may absorb only the ultraviolet, which is ~7% of the sunlight spectrum. Anyway, by appropriate application of CuO/Cu_2_O with direct band gaps, it enables absorption of most of the solar spectrum. As bangaps of CuO/Cu_2_O depend strongly on the deposition method, it is worth noting that it may take values from the range 1.0–2.1 eV for CuO [28,29,30,31,32] and 1.82–2.2 eV [33,34,35,36] for Cu_2_O, respectively. In our research, for CuO structure, we received values from the range 1.02–1.28 [37]. As in the case of multi-layer solar cells, there appears a lattice mismatch according to the materials applied [38,39]; in our analysis, we obtained a lattice mismatch between TiO_2_ and Cu_2_O of ~11%, while in the case of CuO and Cu_2_O, it was ~8.9% [38] (for TiO_2_/Cu_2_O, Hussain et al. [26] reported the vaule of ~25%). In [40] we showed that a lattice mismatch problem revealed a break in the continuity of the crystal structure of samples prepared in our lab.

Pavan et al. [18] investigated a TiO_2_/Cu_2_O all-oxide heterojunction solar cell with the use of the spray pyrolysis method. The authors applied a combinatorial approach and high throughput electrical and optical characterization, and derived a direct band gap of 2.5 eV, which revealed a strong absorption coefficient. The authors showed that almost every photon with energy above 2.5 eV can be absorbed within a Cu_2_O layer (300 nm thick).

The highest conversion efficiency for TiO_2_/CuO solar cells, 1.62%, was reported by Rokhmat et al. [19]. The authors reached that by depositing copper particles using fix-current electroplating and with a copper particle content of 7.93% mass.

Patel et al. [22] produced Ag/CuO_x_/TiO_2_/FTO solar cells, with a p–n junction of 150 nm. The authors investigated several devices, with different oxygen vacancies in CuO_x_, and studied how it influences the performance of cells. They showed that thanks to average transmittance of 28–52%, such devices become transparent for the visible spectrum of the solar light, and semitransparent for the visual notion. The solar cell characteristics were carried out for the UV light of 365 nm, and revealed a linear increase in V_oc_ (open circuit voltage) from 0.266 to 1.01 V, and the change in CuO_x_ phases from Cu_2_O to CuO was observed. The best solar cell with the CuO as an active layer was obtained for the following parameters: open circuit voltage—1.01 V, current density—3.48 mA/cm^2^, and fill factor—31%. Such a combination resulted in the overall efficiency of 16.22% (for an input power of 7 mW/cm^2^). The authors also investigated the influence of TiO_2_ as a buffer layer and showed that the CuO/TiO_2_ device ensures the highest efficiency due to large conduction band offset. Cu_2_O and TiO_2_ were applied in dye-sensitized solar cells (DSSCs), which also are promising solar devices with high effiency [41,42,43,44,45,46,47]. Ursu et al. [43] have investigated p-type DSSC based on full Cu_2_O electrodes with the use of traditional dye and a redox mediator. The authors prepared the counter electrode and the semiconductor material for the cathode based on Cu_2_O in single low-temperature hydrothermal technology. Such a structure provided twice as much JSC and VOC as a DSSC with the use of platinum as a counter electrode. By replacing conventional Pt with a Cu_2_O/Cu counter electrode, the power conversion efficiency was improved by app. 60%. Baptayev et al. [47] considered In-doped TiO_2_ photoanode for DSSC, prepared with the use of a simple surface-doping technique by soaking the TiO_2_ film in acidic In^3+^ solution at 70 °C in a different time period, followed by sintering at 450 °C. Structural characterization of such a structure revealed the successful attachment of indium to the surface of TiO_2_ films and showed that the amount of In dopant was proportional to the soaking time. The authors reported that photoelectric conversation efficiency of such doped devices, for a soaking time of 30 min, resulted in an increase of 18.0% compared to the cells without doping. Indium doping resulted in the downward shift of the TiO_2_ conduction band as well as a decrease in electron recombination, which caused the significant increase in the Voc and fill factor values.

It is also worth mentioning that Cu_2_O was widely studied, e.g., in the context of photoelectrochemical (PEC) cells, which can be applied for solar water splitting [48,49]. Bai et al. [48] showed that Cu_2_O/Ni(OH)_2_, used as a photocathode, results in a larger photocurrent and smaller onset potential compared with Cu_2_O. The authors obtained the photoconversion efficiency at the level of 0.20% under one sun illumination. Chen et al. [49] considered a dual Cu_2_O cell and showed that the deposition of the TiO_x_ overlayer on the Cu_2_O supplies an additional photovoltage, which makes the hydrogen evolution reaction proceed faster. For the tandem device of n-TiO_x_/p-Cu_2_O//n-Cu_2_O, the authors obtained the solar-to-hydrogen efficiency of 0.32%.

Yet another application of TiO_2_ and Cu_x_O can be found in the context of perovskite solar cells [50,51,52,53,54,55,56]. TiO_2_ is used as an electron transport layer (ETL), while Cu_x_O is used as a hole transport layer (HTL). Zhu et al. [51] applied magnetron sputtering technology on FTO glass to create different thicknesses and shapes of TiO2 ETLs, and based on this technology, prepared planar heterojunction perovskite solar cells. The authors observed a decrease in the surface roughness of the TiO2 film together with an increase in the sputtering time, while the electrical homogeneity and square resistance of the film increased. They obtained the maximum film transmittance value of 82.29% and the highest photoelectric conversion efficiency (PCE) of perovskite solar cells of 12.42%. Chen et al. [55] examined perovskite solar cells based on CuO-Cu_2_O thin films, fabricated on ITO-coated glass. The authors applied a CH_3_NH_3_PbI_3_ perovskite absorber on top of a CuO-Cu_2_O structure with the use of a one-step spin-coating process with a toluene washing treatment. By applying different temperatures and thicknesses of Cu-Cu_2_O film, they reached the maximum PCE value of 8.1% and concluded that such a structure is a good candidate for alternative HTL in large-scale perovskite solar cell production.

Among many heterojunction solar cells being considered in literature (see e.g., [57,58,59,60,61,62,63]), our group deals with TiO_2_/Cu_x_O and we already published several papers with investigations of TiO_2_ and Cu_x_O cells fabricated with the direct current magnetron sputtering (DC-MS) with different process conditions [38,40,64,65,66]. In [64] TiO_2_, Cu_2_O, as well as TiO_2_/Cu_2_O, structures were investigated, with the wide variety of measurements carried out: morphology, cross-section, topography, roughness, and transmission spectra of the films. Despite the selection of different parameters, no photovoltaic effect was detected. The first success of obtaining the TiO_2_/CuO solar cell was reported in [65]. The authors improved the performance of TiO_2_ and CuO structures by increasing the oxygen flow rates and substrate temperature during the deposition of the cells mentioned above, compared with [64]. It allowed us to obtain the efficiency of 0.24%. In 2021, again by applying different parameters, such as substrate temperature, oxygen and argon flow rates, as well as time deposition for the TiO_2_/Cu_2_O/CuO/Cu_2_O cell, the authors obtained the efficiency of 0.9%, which is the best result so far [38]. In [66], the authors investigated TiO_2_/CuO/Cu_2_O solar cells with the following measurements: X-ray diffraction, scanning electron microscopy, and current–voltage characteristics. The main goal was to check how the deposition times of the top contact point of Cu and the TiO_2_ layer influence the structural and electrical properties of solar cells being investigated. Such an approach did not result in detecting expected efficiency. The paper [40] contains an analysis of the TiO_2_:ZnO/CuO structure performance depending on the Cu diffusion in the layer formation process. It was shown that the presence of a Cu buffer layer, as well as a smooth start to the deposition procedure of the CuO layer, play a crucial role in the cell production procedure. Investigations revealed that the high resistance and inconsistency must be eliminated to improve the parameters of the structure.

In the present paper, with the use of the direct current magnetron sputtering (DC-MS) method, the authors investigate the influence of different parameters, such as layer thickness and sputtering time, on the properties and efficiency of TiO_2_ and Cu_x_O thin film layers.

## 2. Materials and Methods

TiO_2_/Cu_x_O thin film solar cells were deposited using the direct current (DC) reactive magnetron sputtering method.

Titanium dioxide and copper oxide thin films were sputtered by two metallic targets: Ti (99.995% purity) and Cu (99.995% purity), respectively, with a diameter of each 25.4 mm (Kurt J. Lesker Company, Leonards-On-Sea, UK) on commercial silicon plates (N-type Si, 100) or glass-coated indium-tin oxide (ITO 100 × 100 × 1.1 mm, Kavio).

Initially, the n-type TiO_2_ emitter layer was deposited on substrates for 25 min or 24 min (for the sample #38) in a mixture of argon: oxygen (Ar:O_2_) with a ratio of 1:2.5. The magnetron was powered by 100 W.

Next, a thin film with Cu buffer was deposited for 5 s (#28) or 7 s (samples #29, #32, #35, #37, and #38) for each sample with argon flow rates of only 4 cm^3^/s using a Cu target. Then, the magnetron shutter was closed, flows were set for deposition of the Cu_x_O layer, the plasma beam was stabilised for 20 s (samples #28, #32), 30 s (samples #29, #38), or 60 s (samples #35, #37) with the shutter closed, and the p-type Cu_x_O absorber layers were grown. A mixed atmosphere Ar:O_2_w had a ratio of 1:3.5 and the magnetron was powered by 70 W.

**Figure 1:**
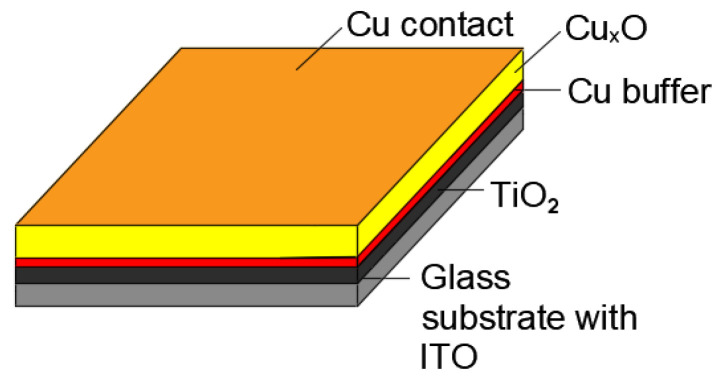
Scheme of the sputtered layers TiO_2_, Cu buffer, Cu_x_O, and Cu contact.

For the thin films of TiO_2_ and Cu_x_O, the pressure during deposition was kept at ~1 × 10^−2^ mbar, the distance between the substrate, the target was equal to 58 mm, and the substrate temperature during thin film deposition was kept at 300 °C. Finally, a thin Cu film was deposited on top of the Cu_x_O as a contact. Details of the sputtering conditions and values of the thickness of the TiO_2_ and Cu_x_O layers are listed in Table 1 and Table 2. A scheme of the TiO_2_/Cu_x_O thin film heterostructure is shown in Figure 1.

To create PV devices, two Cu electrodes were attached to the upper Cu contacts and the ITO by Ag (silver) conductive glue.

The authors invite readers to view a YouTube video of the PREVAC apparatus and PVD laboratory, which are linked in the Supplementary Materials section of this paper.

## 3. Results

### 3.1. Cross Section Analysis of TiO_2_/Cu_x_O Thin Films

**Figure 2:**
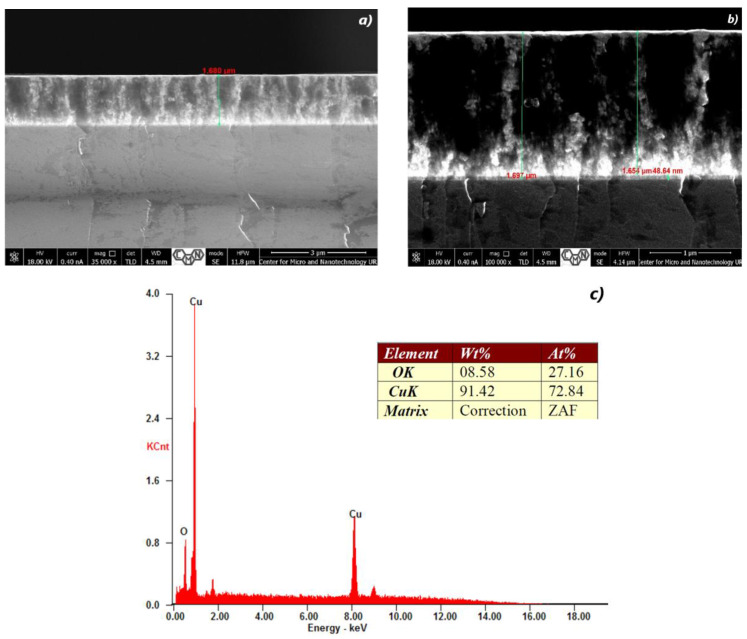
The SEM cross-section image (**a**,**b**) of TiO_2_/Cu_x_O layers and EDX spectra of the sample #28 (**c**).

**Figure 3:**
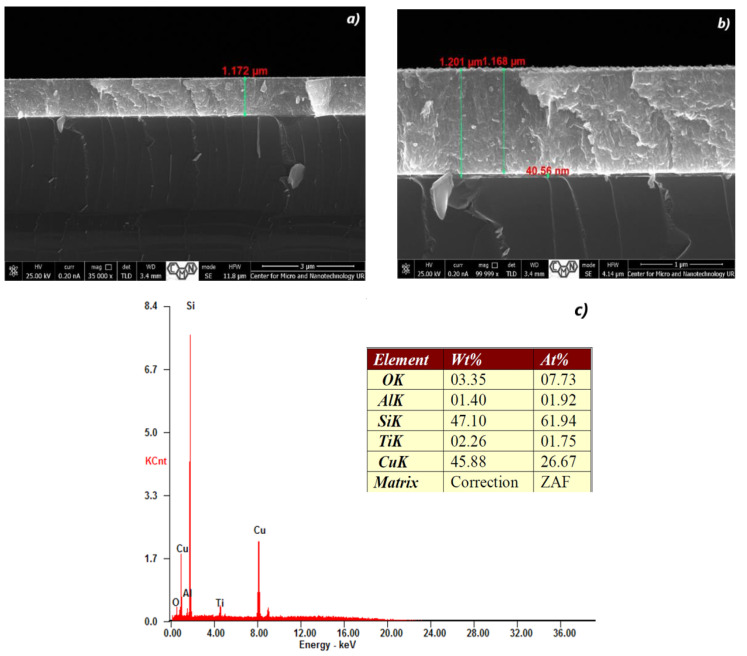
The SEM cross-section image (**a**,**b**) of TiO_2_/Cu_x_O layers and EDX spectra (**c**) of the sample #29.

**Figure 4:**
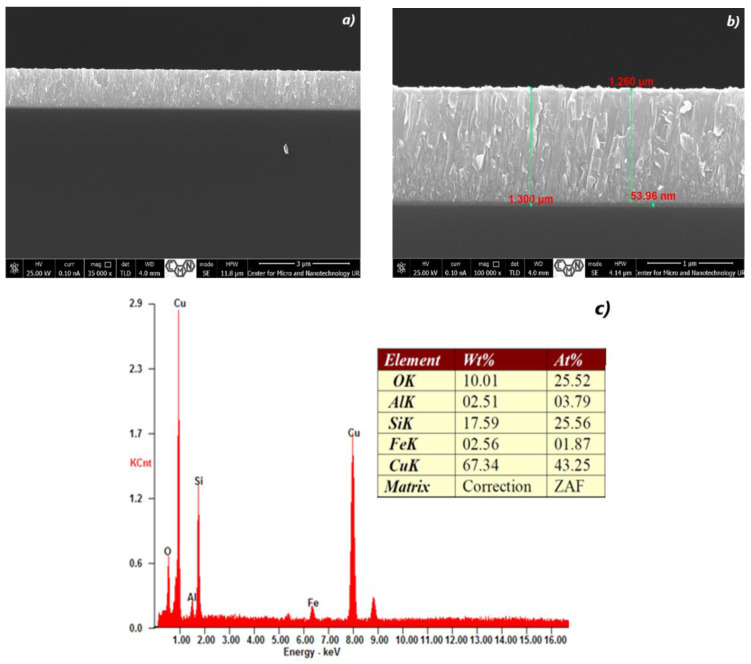
The SEM cross-section image (**a**,**b**) of TiO_2_/Cu_x_O layers and EDX spectra (**c**) of the sample #32.

**Figure 5:**
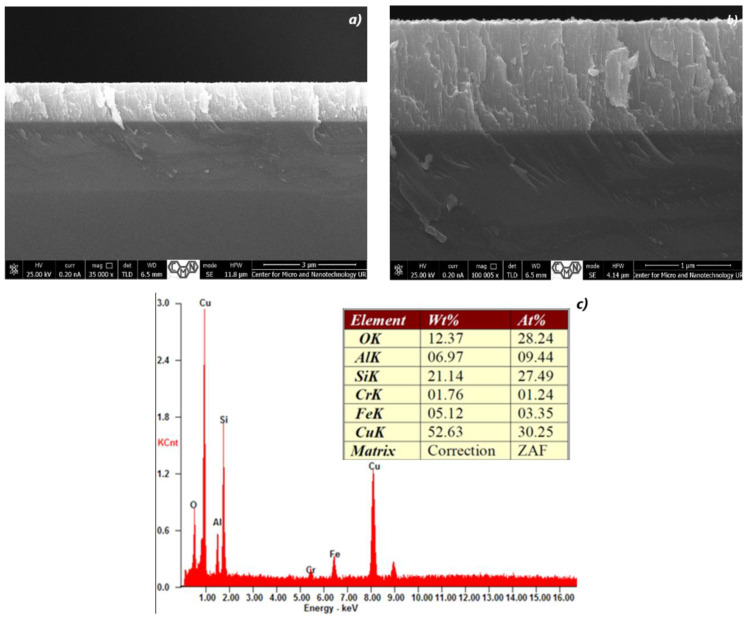
The SEM cross-section image (**a**,**b**) of TiO_2_/Cu_x_O layersand EDX spectra (**c**) of the sample #35.

**Figure 6:**
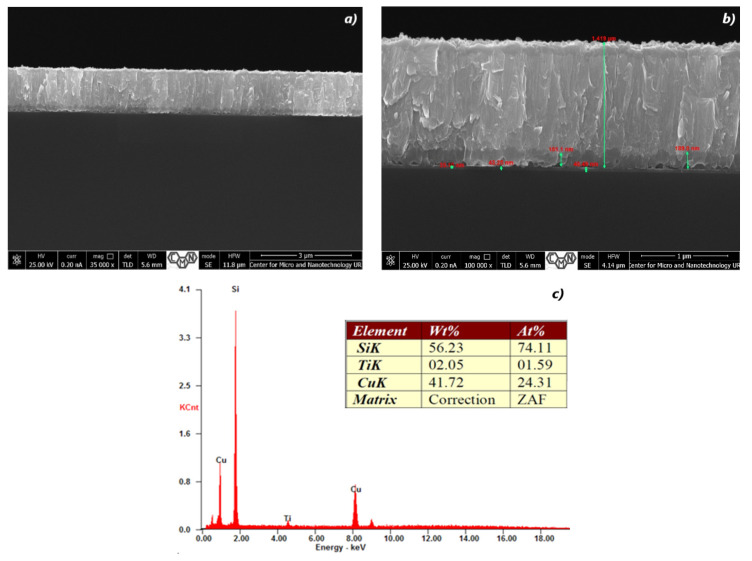
The SEM cross-section image (**a**,**b**) of TiO_2_/Cu_x_O layers and EDX spectra (**c**) of the sample #37.

**Figure 7:**
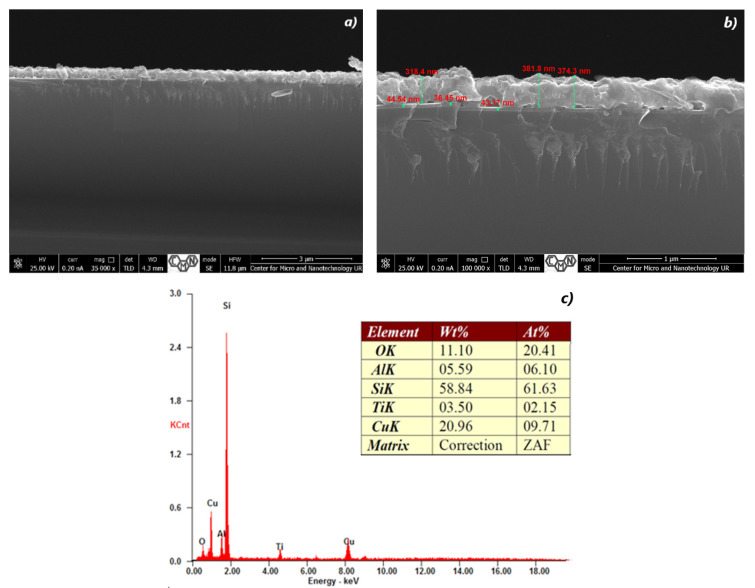
The SEM cross-section image (**a**,**b**) of TiO_2_/Cu_x_O layers and EDX spectra (**c**) of the sample #38.

Figure 2, Figure 3, Figure 4, Figure 5, Figure 6 and Figure 7 show the cross-section of TiO_2_/Cu_x_O layers deposited on (Si) silicon substrates according to the growth parameters shown in Table 1 and Table 3. It is obvious that the films have a similar structure. In the case of CuO, small TiO_2_ clusters were formed on the CuO surface.

Cu_x_O forms a fine-grained film (Figure 2a,b) with a non-monotonic distribution of these crystallites in thickness; the structure resembles a fibrous one, as evidenced by the alternation of dark and light areas in the images. Higher density is characteristic of the initial layers, and the following conglomerates are grouped in the vertical direction of film growth.

For sample #28 (Figure 2a,b), it should be noted that the thickness of 1654 nm of the CuO layer is the largest. The structure of the film is more uniform and completed its forming; without clearly defined faces, the columns of the film grow in different directions. The clearly observed grains are already present in Figure 3a,b, and the growth of individual conglomerates is observed. These are areas with a uniform structure and orientation of atoms. The sizes of such grains vary from 20 to 960 nm. The formation of grains occurs through the processes of nucleation and growth. A small number of nuclei or germs of grains is formed randomly in the process of nucleation [67,68]. These nuclei can be formed as a result of thermal fluctuations or other external influences. After the formation of nuclei, grains begin to grow through the diffusion of material inside the nucleus from the core to the margins. This process leads to an increase in grain size and the development of its crystal structure. Grain growth can occur by various mechanisms, such as diffusion growth, grain boundary migration, etc. Factors that affect the formation of grains in a thin film include the chemical composition of the material, temperature, growth time, deposition, or growth rate, atmospheric conditions, and other production parameters [69].

Elemental or chemical analysis was performed with EDX using the SEM approach. The EDX spectrum for samples #28, #29, #32, #35, #37, and #38 of different thicknesses is shown in Figure 2, Figure 3, Figure 4, Figure 5, Figure 6 and Figure 7. The EDX analysis was carried out in a low vacuum mode. In particular, the percentages of atomic weight are Cu (72.84%) and O (27.16%) for sample 28 (Figure 1c).

For all other samples (Figure 2, Figure 3, Figure 4, Figure 5 and Figure 6), a high percentage contribution of silicon atoms, Si, as the main element of the substrate was observed in the spectrum. Al and Fe atoms are present in the spectrum as the material of the installation table on which the sample was placed, but their contribution is not significant and should be neglected.

The content of 1.75% Ti is sufficient for the film structure to resemble a lamellar structure (Figure 3), and for the surface to be rougher. When such films are split in the transverse direction, the individual plates are separated parallel to the fault plane.

With an increase in the Cu content to 43.25% and the absence of Ti (Figure 4c), the size of the plates decreases significantly; they are randomly arranged (Figure 4a,b). Only a significant surface roughness remains.

At a Cu content of 30.25%, and with the absence of Ti (Figure 5c), the lamellar structure is preserved with the orientation of the planes in the direction perpendicular to the sample surface (Figure 5a,b). The surface roughness of such a film is close to the morphology of the sample #29.

In Figure 6 and Figure 7, thin film island growth is a process in which a thin film is grown on a substrate or layer while maintaining structural correspondence with the substrate. During this process, atoms or molecules of the film material diffuse or deposit on the surface of the substrate, forming a new film layer. Diffusion processes regulate the homogeneity of the film in the lateral direction. Transport from the vapor phase (layer growth) controls the homogeneity of the film in the normal direction [66]. The formation of a uniform film structure can occur only when, in the process of growth, atoms can freely pass between the layers of the film. This means that it is energetically more profitable for an atom that can settle on the top of the island to move to the lower layer, where the potential energy is lower [70].

The presence of Ti (1.6%) leads (Figure 6c) to the formation of hollow areas and introduces randomness in the orientation of lamellar crystallites (Figure 6a,b). The surface of such a film is more irregular and has a more complex relief. The conglomerate structure is preserved, but it becomes more disordered.

An increase in the amount of Ti to 2.15% leads to the separation of Cu_x_O crystallites (Figure 7c), enlargement of their size, and compaction of the material to form more massive crystallites (Figure 7a,b). In general, there is an increase in the number and size of cavities between the substrate and the film.

### 3.2. Solar Cell Performance

**Figure 8:**
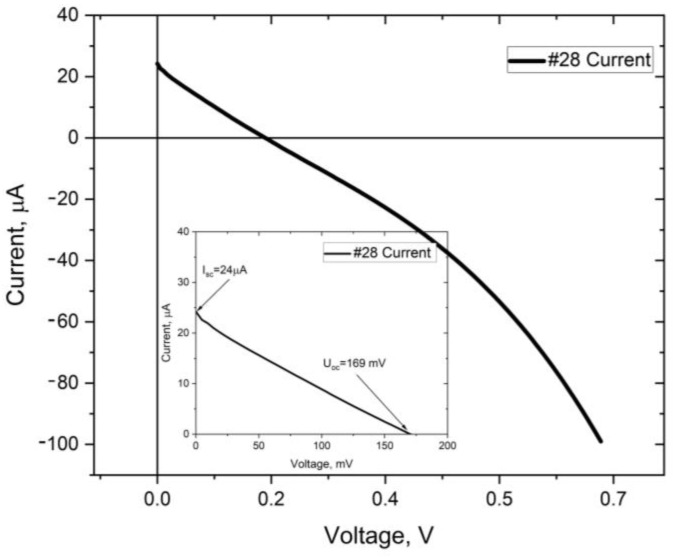
Voltage–current and power–current characteristics of the #28 solar cell.

**Figure 9:**
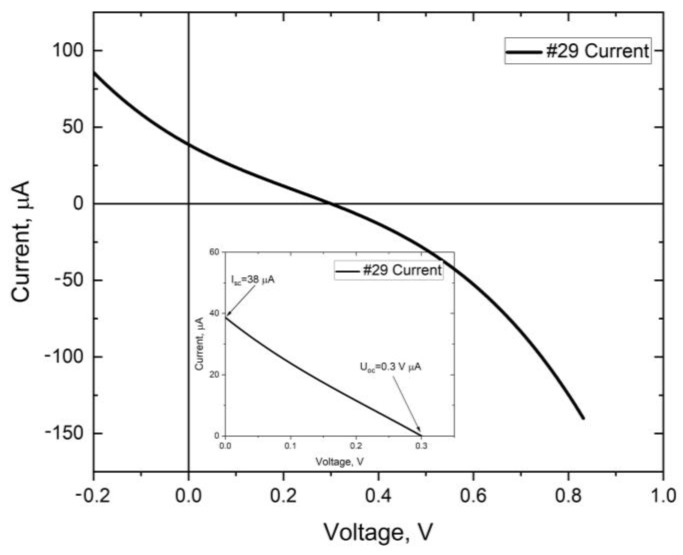
Voltage–current and power–current characteristics of the #29 solar cell.

As the final verification of the proposed concept, complete photovoltaic structures were tested according to their opto-electrical parameters. I-V characteristics were measured under AM 1.5 spectrum in STC conditions by a Quantum Design solar simulator. Table 3 presents specific values achieved for various samples, which demonstrate a photovoltaic effect. For one of the most promising samples (#28 and #29), I-V and P-V characteristics are presented in Figure 8 and Figure 9, respectively. In Table 3, the comparison of electrical parameters for the whole set of samples is demonstrated.

For better understanding of the sample’s parameters, the shunt resistance (Rsh) and series resistance (R_s_) of two selected cells with the best parameters were calculated and presented separately in Table 4.

## 4. Discussion

The Stranski–Krastanov (layer plus island) growth mode was observed in samples #28 and #29. The general feature of layer plus island growth mode is that after the completion of the formation of the two-dimensional layer, the growth of three-dimensional islands takes place. The thickness and orientation of islands are very different in lateral and normal directions. For the sample #28, after 30 min of the deposition process, the film thickness of CuO reached 1654 nm (Table 2), and for the TiO_2_ layer, 49 nm was deposited during 25 min. The next sample, #29, was obtained with a shorter deposition time of 22 min and accordingly reached the thickness of 1201 nm, and for the TiO_2_ layer, 41 nm was deposited during 25 min. Thus, this mode is most often observed in heteroepitaxial thin films, which leads to a change in physical properties and has important applications in semiconductor systems [71,72].

The basis responsible for the structure of thin film formation is the processes of nucleation and growth. It is generally accepted that in the simplest cases, when mutual diffusion does not occur, three growth models are possible. In some cases, the bond (with energy E_b_) of condensing adatoms is much stronger among themselves than with the substrate (E_a_) [73,74,75,76,77,78]. As a rule, when the energy E_b_ < E_a_ adatoms begin to diffuse with low diffusion energy, which leads to fusion with other adatoms, at the same time, small stable clusters begin to form. The formed small cluster of adatoms, which are connected to each other by the energy Ei, can often consist of only one atom, which corresponds to the critical stage of nucleation. In this case, the formed pairs of adatoms have energy Ei = 0 and are stable on the thin film surface. In another case, when the energy E_a_ is greater than several E_b_, layer-by-layer growth begins. During such growth, the reevaporation process does not occur, which leads to a linear increase in the concentration of one atom (n_i_). Subsequent nucleation processes are rather more complicated, due to the fact that several modes compete for these adatoms.

Observing the presented characteristics, one may clearly indicate the presence of the photovoltaic effect in the manufactured structures. The general flow of the I-V curves confirm the p-n junction operation, but the I_sc_ and V_oc_ values are limited far below the available theoretical values, and additionally, the fill factor of these devices is even below 25%, which greatly reduces maximum power and efficiency. A falling of the FF value below 25% points out the improper tilt of the characteristics, and is caused by reverse-polarized junction. This fact is especially visible even in the case of the sample #29 with a very low FF value of 16.5%. This phenomenon occurs often in the junction regions, where instead of ohmic contacts, a reverse-polarized Schottky barrier appears. For the record samples (#28 and #29), achievement of FF at the level of 60% would increase the efficiency to the level of 0.032% for #28 and 0.036% for #29, respectfully. This would also result in the power at the level of 7 µW. This fact prompts the contact structure optimization.

For better understanding of the technological process parameters’ influence on the sample performance as well as cell construction importance, the Voc/thickness ratios were calculated for all samples (Table 3). These values were referred to both CuO and TiO_2_ layers. Excluding sample #38 with the extremely low absorber thickness, one may observe that the peak open circuit voltage was present when the low thickness of the window layer (41 nm in the case of sample #29) meets a sufficient, but not too thick, absorber layer (1200 nm in case of sample #29). The second in the efficiency gradation #28 sample presents the thickest absorber and a relatively thick window layer, but the proper crystalline structure is also an important factor in this case.

Considering the influence of the resistance on the device parameters, the two best performance solar cells (samples #28 and #29) were compared. In the cases of both these devices, one may observe relatively high series resistance; which, in the case of the sample #29, is two times higher. This resulted in lower energy production of the sample #29; but in both cases, such high R_s_ values dramatically reduced the FF value. The shunt resistance of the sample #29 is much superior, being several orders of magnitude higher. Summarizing, for the final performance, the value of low series resistance appeared more important for the FF value and the final power gain than limited shunt losses; however, for future development, all these values shall be improved.

## 5. Conclusions

TiO_2_/Cu_x_O thin film solar cells were deposited using the direct current (DC) reactive magnetron sputtering method. From cross-section analysis, it is obvious that the films have a similar structure and growth of individual conglomerates, with grains from 20 to 960 nm. The best crystalline structure of the samples and optimal material composition results in the highest I-V parameters range. Such a growth mechanism corresponds to the Stranski–Krastanov (layer plus island) growth mode. The general feature of this growth mode is that virtually any impediment to continuous layer growth results in the formation of islands on top of (or in some cases within) an intermediate layer, whose thickness and orientation is very different in the different systems. A noticeable photovoltaic effect was achieved in all samples, with the top values of power at the level of a few microwatts. The future optimization towards higher I-V values by an increase in the fill factor value and elimination of a counter-junction effect is planned in the near-future research.

## Figures and Tables

**Table 1:** Growth parameters of the TiO_2_ window layer.

	#28	#29	#32	#35	#37	#38
Time [min]	25					24
Power [W]	100					
Pressure [mbar]	1.15 × 10^−2^	1.05 × 10^−2^	1.11 × 10^−2^	1.08 × 10^−2^	1.06 × 10^−2^	1.11 × 10^−2^
Distance between the source and substrate [mm]	58					
Oxygen flow rate [cm^3^/s]	2.5					
Argon flow rate [cm^3^/s]	1					
Substrate temperature [°C]	300					
Thickness [nm]	49	41	54	43	49	45

**Table 2:** Growth parameters of the CuO absorber layer.

	#28	#29	#32	#35	#37	#38
Time [min]	30	22	25			22
Power [W]	70					
Pressure [mbar]	1.11 × 10^−2^	1.11 × 10^−2^	1.14 × 10^−2^	1.11 × 10^−2^	1.11 × 10^−2^	1.10 × 10^−2^
Distance between the source and substrate [mm]	58					
Oxygen flow rate [cm^3^/s]	3.5					
Argon flow rate [cm^3^/s]	1					
Substrate temperature [°C]	300					
Thickness [nm]	1654	1201	1260	1305	1419	318

**Table 3:** Basic I-V parameters of the manufactured samples.

	#28	#29	#32	#35	#37	#38
V_oc_ [mV]	176	300	75	14	23	71
Isc [µA]	21	38	4.3	7	1.5	5.3
µ [%]	0.01	0.01				
Contact area [mm^2^]	30	35	45		36	40
V_oc_/CuO thickness [mV/nm]	0.10	0.25	0.06	0.01	0.02	0.22
V_oc_/TiO2 thickness [mV/nm]	3.59	7.32	1.39	0.33	0.47	1.58

**Table 4:** Series (R_s_), shunt resistance (R_sh_), and fill factor value of the two selected samples.

	#28	#29
R_s_ [Ω]	27 k	55 k
R_sh_ [Ω]	6.2 k	270 M
FF [%]	18.5	16.6

## Data Availability

The data presented in this study are available on request from the corresponding author.

## Supplementary Materials

The Supplementary information can be found at: Link I: https://www.youtube.com/watch?v=-0Sn4UbiKaE (accessed on 28 June 2023); Link II: https://www.youtube.com/watch?v=Lavsm1CIqhY (accessed on 28 June 2023); Link III: https://www.youtube.com/watch?v=iei5bn2UAzg\046t=35s (accessed on 28 June 2023); Link IV: https://www.youtube.com/watch?v=0TjWJwxLZYk\046t=4s (accessed on 28 June 2023).

## References

[1] Song X., Ji X., Li M., Lin W., Luo X., Zhang H. (2014). A Review on Development Prospect of CZTS Based Thin Film Solar Cells. Int. J. Photoenergy.

[2] GuGuo H., Ma C., Zhang K., Jia X., Wang X., Yuan N., Ding J. (2018). Dual function of ultrathin Ti intermediate layers in CZTS solar cells: Sulfur blocking and charge enhancement. Sol. Energy Mater. Sol. Cells.

[3] Kumar A., Thakur A.D. (2018). Role of contact work function, back surface field, and conduction band offset in Cu_2_ZnSnS_4_ solar cell. Jpn. J. Appl. Phys..

[4] Lee D., Yang J.Y. (2020). Superstrate structured FTO/TiO_2_/In_2_S_3_/Cu_2_ZnSnS_4_ solar cells fabricated by a spray method with aqueous solutions. Coatings.

[5] Satale V.V., Bhat S.V. (2020). Superstrate type CZTS solar cell with all solution processed functional layers at low temperature. Sol. Energy.

[6] Khalil M.I., Bernasconi R., Lucotti A., Le Donne A., Mereu R.A., Binetti S., Hart J.L., Taheri M.L., Nobili L., Magagnin L. (2021). CZTS thin film solar cells on flexible Molybdenum foil by electrodeposition-annealing route. J. Appl. Electrochem..

[7] Li J., Sun K., Yuan X., Huang J., Green M.A., Hao X. (2023). Emergence of flexible kesterite solar cells: Progress and perspectives. Npj Flex. Electron..

[8] Shockley W., Queisser H.J. (1961). Detailed balance limit of efficiency p-n junction solar cells. J. Appl. Phys..

[9] Hernández-Gutiérrez C.A., Morales-Acevedo A., Cardona D., Contreras-Puente G., López-López M. (2019). Analysis of the performance of InxGa1−xN based solar cells. SN Appl. Sci..

[10] Jose R., Velmurugan T., Seeram R. (2009). Metal oxides for dye-sensitized solar cells. J. Am. Ceram. Soc..

[11] Joni H., Jyrki L., Jarkko P., Tomi H., Anita S., Samuli K. (2015). Pulsed laser deposition of metal oxide nanoparticles, agglomerates, and nanotrees for chemical sensors. Proc. Eng..

[12] Ho S.M. (2016). Synthesis and properties of cadmium oxide thin films: A review. Int. J. Curr. Adv. Res..

[13] Son M.-K., Steier L., Schreier M., Mayer M.T., Luo J., Grätzel M. (2017). A copper nickel mixed oxide hole selective layer for Au-free transparent cuprous oxide photocathodes. Energy Environ. Sci..

[14] Pérez-Tomás A., Mingorance A., Tanenbaum D., Lira-Cantú M., Lira-Cantú M. (2018). Chapter 8—Metal Oxides in Photovoltaics: All-Oxide, Ferroic, and Perovskite Solar Cells. Metal Oxides Series: The Future of Semiconductor Oxides in Next-Generation Solar Cells.

[15] He H., Cui Z., Korotcenkov G. (2020). 2—Metal oxide semiconductors and conductors. Metal Oxide Series: Solution Processed Metal Oxide Thin Films for Electronic Applications.

[16] Horta I.M., Godoy A., Damasceno B.S., de Jesus Pereira D.L., Leite D.M.G., da Silva Sobrinho A.S., Kumar N., Soucase B.M. (2023). 10—Development of metal oxide heterostructures for photovoltaic and solar cell applications. Metal Oxide-Based Heterostructures Fabrication and Applications.

[17] Lee Y.S., Chua D., Brandt R.E., Siah S.C., Li J.V., Mailoa J.P., Lee S.W., Gordon R.G., Buonassisi T. (2014). Atomic layer deposited gallium oxide buffer layer enables 1.2 V open-circuit voltage in cuprous oxide solar cells. Adv. Mater..

[18] Pavan M., Rühle S., Ginsburg A., Keller D.A., Barad H.-N., Sberna P.M., Nunes D., Martins R., Anderson A.Y., Zaban A. (2015). TiO_2_/Cu_2_O all-oxide heterojunction solar cells produced by spray pyrolysis. Sol. Energy Mater. Sol. Cells.

[19] Rokhmat M., Wibowo E., Sutisna K., Abdullah M. (2017). Performance improvement of TiO_2_/CuO solar cell by growing copper particle using fix current electroplating method. Procedia Eng..

[20] Iivonen T., Heikkilä M.J., Popov G., Nieminen H.-E., Kaipio M., Kemell M., Mattinen M., Meinander K., Mizohata K., Räisänen J. (2019). Atomic layer deposition of photoconductive Cu_2_O thin films. ACS Omega.

[21] Wojcieszak D., Obstarczyk A., Domaradzki J., Kaczmarek D., Zakrzewska K., Pastuszek R. (2019). Investigations of structure and electrical properties of TiO_2_/CuO thin film heterostructures. Thin Solid Films.

[22] Patel D.B., Chauhan K.R. (2020). 50% transparent solar cells of CuO_x_/TiO_2_: Device aspects. J. Alloys Compd..

[23] Sawicka-Chudy P., Sibiński M., Rybak-Wilusz E., Cholewa M., Wisz G., Yavorskyi R. (2020). Review of the development of copper oxides with titanium dioxide thin film solar cells. AIP Adv..

[24] Ghrib T., AL-Saleem N.K., AL-Naghmaish A., Elshekhipy A.A., Brini S., Briki K., Elsayed K.A. (2022). Annealing effect on the microstructural, optical, electrical, and thermal properties of Cu_2_O/TiO_2_/Cu_2_O/TiO_2_/Si heterojunction prepared by sol-gel technique. Micro Nanostr..

[25] Sharma A.K., Tareja R.K., Wilker U., Schade W. (2003). Phase transformation in room temperature pulsed laser deposited TiO2 thin films. Appl. Surf. Sci..

[26] Hussain S., Cao C., Usman Z., Chen Z., Nabi G., Khan W.S., Ali Z., Butt F.K., Mahmood T. (2012). Fabrication and photovoltaic characteristics of Cu_2_O/TiO_2_ thin film heterojunction solar cell. Thin Solid Films.

[27] Dette C., Pérez-Osorio M.A., Kley C.S., Punke P., Patrick C.E., Jacobson P., Giustino F., Jung S.J., Kern K. (2014). TiO_2_ anatase with a bandgap in the visible region. Nano Lett..

[28] Xu L., Zheng G., Pei S., Wang J. (2018). Investigation of optical bandgap variation and photoluminescence behavior in nanocrystalline CuO thin films. Optik.

[29] Tripathi T.S., Terasaki I., Karppinen M. (2016). Anomalous thickness-dependent optical energy gap of ALD-grown ultra-thin CuO films. J. Phys. Condens. Matter.

[30] Zheng W., Chen Y., Peng X., Zhong K., Lin Y., Huang Z. (2018). The phase evolution and physical properties of binary copper oxide thin films prepared by reactive magnetron sputtering. Materials.

[31] Diachenko O., Kováč J., Dobrozhan O., Novák P., Kováč J., Skriniarova J., Opanasyuk A. (2021). Structural and optical properties of CuO thin films, synthesized using spray pyrolysis method. Coatings.

[32] Lakshmanan A., Alex Z.C., Meher S.R. (2022). Recent advances in cuprous oxide thin film based photovoltaics. Mater. Today Sustain..

[33] Ichimura M., Kato Y. (2013). Fabrication of TiO_2_/Cu_2_O heterojunction solar cells by electrophoretic deposition and electrodeposition. Mat. Sci. Semicon. Proc..

[34] Tahir D., Tougaard S. (2012). Electronic and optical properties of Cu, CuO and Cu_2_O studied by electron spectroscopy. J. Phys. Condens. Matter.

[35] Lakshmanan A., Alex Z.C., Meher S.R. (2022). Cu_2_O thin films grown by magnetron sputtering as solar cell absorber layers. Mat. Sci. Semicon. Proc..

[36] Pagare P.K., Torane A.P. (2016). Band gap varied cuprous oxide (Cu_2_O) thin films as a tool for glucose sensing. Microchim. Acta.

[37] Wisz G., Sawicka-Chudy P., Wal A., Potera P., Bester M., Płoch D., Sibiński M., Cholewa M., Ruszała M. (2022). TiO_2_:ZnO/CuO thin film solar cells prepared via reactive direct-current (DC) magnetron sputtering. Appl. Mater. Today.

[38] Wisz G., Sawicka-Chudy P., Sibiński M., Starowicz Z., Płoch D., Góral A., Bester M., Cholewa M., Woźny J., Sosna-Głębska A. (2021). Solar cells based on copper oxide and titanium dioxide prepared by reactive direct-current magnetron sputtering. Opto-Electron. Rev..

[39] Bremner S.P., Yi C., Almansouri I., Ho-Baillie A., Green M.A. (2016). Optimum band gap combinations to make best use of new photovoltaic materials. Sol. Energy.

[40] Wisz G., Sawicka-Chudy P., Wal A., Sibiński M., Potera P., Yavorskyi R., Nykyruy L., Płoch D., Bester M., Cholewa M. (2023). Structure defects and photovoltaic properties of TiO_2_:ZnO/CuO solar cells prepared by reactive DC magnetron sputtering. Appl. Sci..

[41] Sharma K., Sharma V., Sharma S.S. (2018). Dye-sensitized solar cells: Fundamentals and current status. Nanoscale Res. Lett..

[42] Tsai C.H., Fei P.H., Chen C.H. (2015). Investigation of coral-like Cu_2_O nano/microstructures as counter electrodes for dye-sensitized solar cells. Materials.

[43] Baptayev B., Salimgerey A., Balanay M.P. (2020). Surface modification of TiO_2_ photoanodes with In^3+^ using a simple soaking technique for enhancing the efficiency of dye-sensitized solar cells. J. Photochem. Photobiol. A Chem..

[44] Gao F., Wang Y., Shi D., Zhang J., Wang M., Jing X., Humphry-Baker R., Wang P., Zakeeruddin S.M., Gratzel M. (2008). Enhance the optical absorptivity of nanocrystalline TiO_2_ film with high molar extinction coefficient ruthenium sensitizers for high performance dye sensitized solar cells. J. Am. Chem. Soc..

[45] Zhao Y.L., Song D.M., Qiang Y.H., Gu X.Q., Zhu L., Song C.B. (2014). Dye-sensitized solar cells based on TiO_2_ hollow spheres/TiO_2_ nanotube array composite films. Appl. Surf. Sci..

[46] Huang Y., Wu H., Yu Q., Wang J., Yu C., Wang J., Gao S., Jiao S., Zhang X., Wang P. (2018). Single-layer TiO_2_ film composed of mesoporous spheres for high-efficiency and stable dye-sensitized solar sells. ACS Sustain. Chem. Eng..

[47] Ursu D., Vajda M., Miclau M. (2019). Investigation of the p-type dye-sensitized solar cell based on full Cu_2_O electrodes. J. Alloys Compd..

[48] Bai Z., Liu J., Zhang Y., Huang Z., Gao Y., Li X., Du Y. (2020). Unassisted solar water splitting using a Cu_2_O/Ni(OH)_2_-ZnO/Au tandem photoelectrochemical cell. J. Solid State Electr..

[49] Chen Y.C., Yang Z.-L., Hsu Y.-K. (2023). Unassisted solar water splitting by dual Cu_2_O–based tandem device with complementary wavelength–dependent quantum efficiency and antipodal conductivity. Renew. Energy.

[50] Shahvaranfard F., Li N., Hosseinpour S., Hejazi S., Zhang K., Altomare M., Schmuki P., Brabec C.J. (2022). Comparison of the sputtered TiO_2_ anatase and rutile thin films as electron transporting layers in perovskite solar cells. Nanoselect.

[51] Zhu H., Zhang T.-H., Wei Q.-Y., Yu S.-J., Gao H., Guo P.-C., Li J.-K., Wang Y.-X. (2022). Preparation of TiO_2_ electron transport layer by magnetron sputtering and its effect on the properties of perovskite solar cells. Energy Rep..

[52] Zhang X., Zhang Y., Wang Y., Wang Q., Liu Z., Geng R., Wang H., Jiang W., Ding W. (2022). Improving the performance of perovskite solar cells via TiO_2_ electron transport layer prepared by direct current pulsed magnetron sputtering. J. Alloys Compd..

[53] Valastro S., Smecca E., Mannino G., Bongiorno C., Fisicaro G., Goedecker S., Arena V., Spampinato C., Deretzis I., Dattilo S. (2023). Preventing lead leakage in perovskite solar cells with a sustainable titanium dioxide sponge. Nat. Sustain..

[54] Sun W., Li Y., Ye S., Rao H., Yan W., Peng H., Li Y., Liu Z., Wang S., Chen Z. (2016). High-performance inverted planar heterojunction perovskite solar cells based on a solution-processed CuOx hole transport layer. Nanoscale.

[55] Chen L.-C., Chen C.-C., Liang K.-C., Chang S.H., Tseng Z.-L., Yeh S.-C., Chen C.-T., Wu W.-T., Wu C.-G. (2016). Nano-structured CuO-Cu_2_O complex thin film for application in CH_3_NH_3_PbI_3_ perovskite solar cells. Nanoscale Res. Lett..

[56] Arjun V., Muthukumaran K.P., Ramachandran K., Nithya A., Karuppuchamy S. (2022). Fabrication of efficient and stable planar perovskite solar cell using copper oxide as hole transport material. J. Alloys Compd..

[57] Minami T., Nishi Y., Miyata T. (2013). Effect of the thin Ga_2_O_3_ layer in n+-ZnO/n-Ga_2_O_3_/p-Cu_2_O heterojunction solar cells. Thin Solid Films.

[58] Minami T., Nishi Y., Miyata T. (2013). High-efficiency Cu_2_O-based heterojunction solar cells fabricated using a Ga_2_O_3_ thin film as n-type layer. Appl. Phys. Express.

[59] Rizi M.T., Abadi M.H.S., Ghaneii M. (2018). Two dimensional modeling of Cu_2_O heterojunction solar cells based-on β-Ga_2_O_3_ buffer. Optik.

[60] Hernández-Gutiérrez C.A., Casallas-Moreno Y.L., Cardona D., Kudriavtsev Y., Santana-Rodríguez G., Mendoza-Pérez R., Contreras-Puente G., Mendez-Garcia V.H., Gallardo-Hernández S., Quevedo-Lopez M.A. (2019). Characterization of n-GaN/p-GaAs NP heterojunctions. Superlattices Microstruct..

[61] Miyata T., Tokunaga H., Watanabe K., Ikenaga N., Minami T. (2020). Photovoltaic properties of low-damage magnetron-sputtered n-type ZnO thin film/p-type Cu_2_O sheet heterojunction solar cells. Thin Solid Films.

[62] Sekkat A., Bellet D., Chichignoud G., Muñoz-Rojas D., Kaminski-Cachopo A. (2022). Unveiling key limitations of ZnO/Cu_2_O all-oxide solar cells through numerical simulations. ACS Appl. Energy Mater..

[63] Mehta M., Avasthi S. (2023). The possibility of gallium oxide (β-Ga_2_O_3_) heterojunction bipolar transistors. Phys. Scr..

[64] Sawicka-Chudy P., Sibiński M., Pawełek R., Wisz G., Cieniek B., Potera P., Szczepan P., Adamiak S., Cholewa M., Głowa Ł. (2019). Characteristics of TiO_2_, Cu_2_O and TiO_2_/Cu_2_O thin films for application in PV devices. AIP Adv..

[65] Sawicka-Chudy P., Wisz G., Sibiński M., Starowicz Z., Głowa Ł., Szczerba M., Cholewa M. (2020). Performance improvement of TiO_2_/CuO by increasing oxygen flow rates and substrate temperature using DC reactive magnetron sputtering method. Optik.

[66] Wisz G., Sawicka-Chudy P., Sibiński M., Płoch D., Bester M., Cholewa M., Woźny J., Yavorskyi R., Nykyruy L., Ruszała M. (2022). TiO_2_/CuO/Cu_2_O Photovoltaic Nanostructures Prepared by DC Reactive Magnetron Sputtering. Nanomaterials.

[67] Venables J.A., Spiller G.D.T., Hanbucken M. (1984). Nucleation and growth of thin films. Rep. Prog. Phys..

[68] Saliy Y.P., Nykyruy L.I., Yavorskyi R.S., Adamiak S. (2017). The Surface Morphology of CdTe Thin Films Obtained by Open Evaporation in Vacuum. J. Nano Electron. Phys..

[69] Panjan P., Drnovšek A., Gselman P., Čekada M., Panjan M. (2020). Review of growth defects in thin films prepared by PVD techniques. Coatings.

[70] Yavorskyi R.S. (2020). Features of optical properties of high stable CdTe photovoltaic absorber layer. Phys. Chem. Solid State.

[71] Venables J.A. (1986). Nucleation and growth processes in thin film formation. J. Vac. Sci. Technol. B.

[72] Wisz G., Sawicka-Chudy P., Yavorskyi R., Potera P., Bester M. (2021). TiO_2_/Cu_2_O heterojunctions for photovoltaic cells application produced by reactive magnetron sputtering. Mater. Today Proc..

[73] Venables J.A. (1983). Nucleation and growth of thin films: Recent progress. Vacuum.

[74] Robins J.L. (1979). Problems and progress in describing quantitatively the development of thin film deposits. Surf. Sci..

[75] Usher B.F., Robins J.L. (1982). The mobility of gold clusters on sodium chloride at temperatures between 123 K and ambient. Thin Solid Films.

[76] Gates A.D., Robins J.L. (1982). Heterogeneous nucleation on cleavage steps: I. Theory. Surf. Sci..

[77] Harsdorff M. (1982). Heterogeneous nucleation and growth of thin films. Thin Solid Films.

[78] Harsdorff M. (1984). The influence of charged point defects and contamination of substrate surfaces on nucleation. Thin Solid Films.

